# Effect of cell culture density on dental pulp-derived mesenchymal stem cells with reference to osteogenic differentiation

**DOI:** 10.1038/s41598-019-41741-w

**Published:** 2019-04-01

**Authors:** Sonoko Noda, Nobuyuki Kawashima, Mioko Yamamoto, Kentaro Hashimoto, Keisuke Nara, Ichiro Sekiya, Takashi Okiji

**Affiliations:** 1 Department of Pulp Biology and Endodontics, Division of Oral Health Sciences, Graduate School of Medical and Dental Sciences, Tokyo Medical and Dental University (TMDU), 1-5-45 Yushima, Bunkyo-ku, Tokyo, 113-8549 Japan; 2 Center for Stem Cell and Regenerative Medicine, Tokyo Medical and Dental University (TMDU), 1-5-45 Yushima, Bunkyo-ku, Tokyo, 113-8510 Japan

## Abstract

Dental pulp stem cells (DPSCs) are a good source for tissue regeneration, however, the number of DPSCs in the pulp tissue is limited. Cell propagation is essential for tissue engineering using DPSCs and the cell culture conditions may affect the properties of DPSCs. The purpose of this study was to analyze the effect of cell culture condition, especially dense culture condition, on the property and differentiation pathway of DPSCs. We cultured DPSCs under sparse (sDPSCs; 5 × 10^3^ cells/cm^2^) or dense (dDPSCs; 1 × 10^5^ cells/cm^2^) conditions for 4 days and compared their properties. The populations of CD73^+^ and CD105^+^ cells were significantly decreased in dDPSCs. Both groups showed multi-differentiation potential, but mineralized nodule formation was enhanced in dDPSCs. The phosphorylation of focal adhesion kinase (FAK) and phosphoinositide 3-kinase (PI3K) proteins was promoted in dDPSCs, and *alkaline phosphatase (ALP)* mRNA expression in dDPSCs was abolished in the presence of pan-PI3K and FAK inhibitors. dDPSCs implanted into mouse bone cavities induced more mineralized tissue formation than sDPSCs and control. These findings indicate that dense culture conditions modified the properties of DPSCs and gave rise to osteogenic-lineage commitment via integrin signaling and suggest that dense culture conditions favor the propagation of DPSCs to be used for mineralized tissue regeneration.

## Introduction

Mesenchymal stem cells (MSCs) derived from various mesenchymal tissues and organs are thought to be a good source for tissue engineering and regenerative medicine^1,2^. Dental pulp tissue contains dental pulp stem cells (DPSCs), which are undifferentiated neural crest-derived MSCs^3^. DPSCs possess high proliferative activity and high potential to differentiate into various cells including neuronal cells, chondroblasts, adipocytes, and osteoblasts^1,4^, suggesting that they are ideal for tissue engineering and regenerative medicine. Promising results of clinical trials to regenerate bone^5,6^ and dental pulp tissue^1,7^ using DPSCs have recently been reported.

One of the advantages of DPSCs as a source for regenerative medicine is that the dental pulp tissue can be obtained from premolars planned to be extracted for orthodontic reasons or unfunctional/unnecessary wisdom teeth and supernumerary teeth, which are usually abrogated as waste^1^. DPSCs are isolated from the dental pulp tissue of adult/permanent teeth, and deciduous teeth also harbor mesenchymal stem cells known as “stem cells from human exfoliated deciduous teeth” (SHEDs)^8,9^. However, there are some disadvantages associated with the use of DPSCs, including the limited volume of pulp tissue. In tissue regeneration using MSCs, their quality and quantity are keys to induce optimal outcomes of tissue regeneration. A sufficient number of stem cells are thus essential for clinical stem cell transplantation, and generally at least 1 × 10^6^ to 10^7^ MSCs are locally applied^2,7^. Since the yield of DPSCs from extracted teeth is limited, it is essential to increase the number of cells by *in vitro* cell culture.

The cell culture conditions may affect the properties of stem cells^10,11^. For example, confluent culture conditions modify the properties of bone marrow stem cells (BMSCs), limiting their capacities to differentiate into multiple lineages and to proliferate^12,13^. DPSCs are reported to maintain an undifferentiated state even upon long-term cultivation^14^, and to be influenced little by the number of passages^15^. However, the association between cell culture conditions and their properties has not been extensively studied. We hypothesized that the density at which DPSCs are cultured influences their differentiation pathway, and evaluated the effects of sparse and dense cell culture conditions on their mesenchymal stem cell marker expression, proliferation, and capacity to differentiate into multiple lineages. We also examined the involvement of integrin signaling in the differentiation of densely cultured DPSCs, since tight cell–cell contacts may induce the activation of integrin signaling. In addition, we investigated the effects of cell culture conditions on their commitment to mineralized tissue-forming cells.

## Results

### MSC marker expression and differentiation capacity

**Figure 1:**
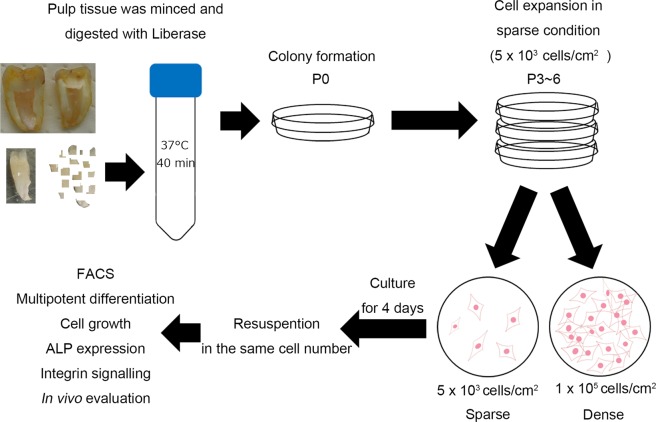
Study scheme. The pulp tissue removed from extracted teeth was minced and digested cells were seeded under sparse conditions. Colony-forming cells (DPSCs) were collected and seeded under sparse conditions (5 × 10^3^ cells/cm^2^) for cell expansion. DPSCs were carefully cultured to maintain their sparsity. Expanded cells (P3–6) were collected and seeded into “sparse” (sDPSCs: 5 × 10^3^ cells/cm^2^) and “dense” groups (dDPSCs: 1 × 10^5^ cells/cm^2^). Following culture for 4 days, cells in both groups were collected and their cell surface markers, multi-differentiation potential, and proliferation were evaluated.

**Figure 2:**
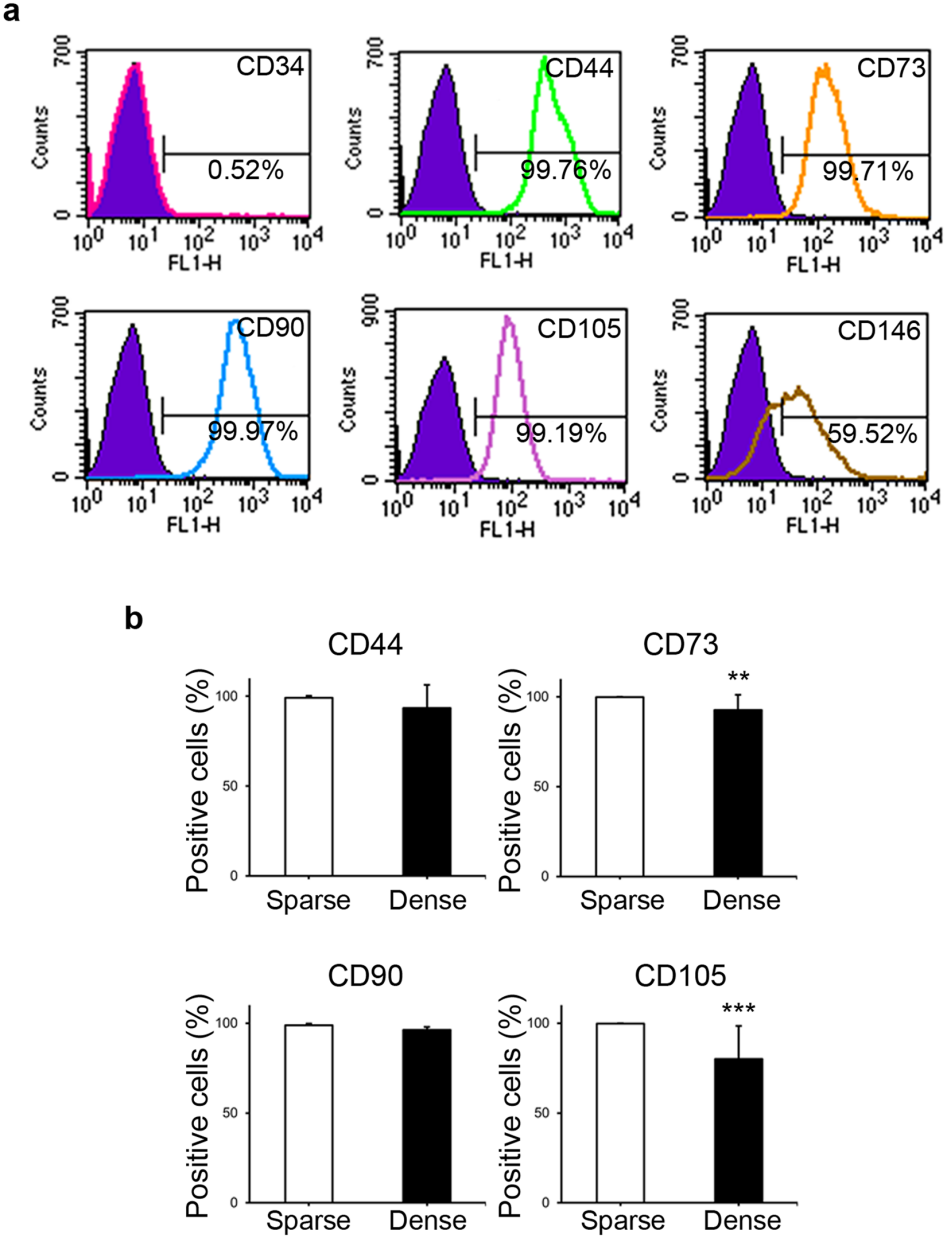
Cell surface markers. (**a**) Cell surface markers before separation into sparse (sDPSCs) and dense (dDPSCs) groups. A representative case among seven donors is shown. (**b**) MSC marker expression in sparse (sDPSCs) and dense (dDPSCs) groups. **p = 0.0079 and ***p = 0.0006 (Mann-Whitney U test). The error bar is SD (n = 7). Solid colored histograms represent IgGκ treated cells for control, and open colored histograms represent fluorophore labeled antibody treated cells.

The experimental scheme is shown in Fig. 1. First, the cell surface marker expression of DPSCs was evaluated prior to their exposure to the “sparse” and “dense” culture conditions. Almost all the cells expressed CD44 (99.17 ± 1.03%; mean ± SD), CD73 (99.90 ± 0.10%), CD90 (98.94 ± 0.74%), and CD105 (99.70 ± 0.24%), and more than half expressed CD146 (61.67 ± 22.84%). In contrast, CD34-expressing cells were rarely observed (1.72 ± 0.85%). A typical case of cell surface marker expression among seven individual samples is shown in Fig. 2a.

**Figure 3:**
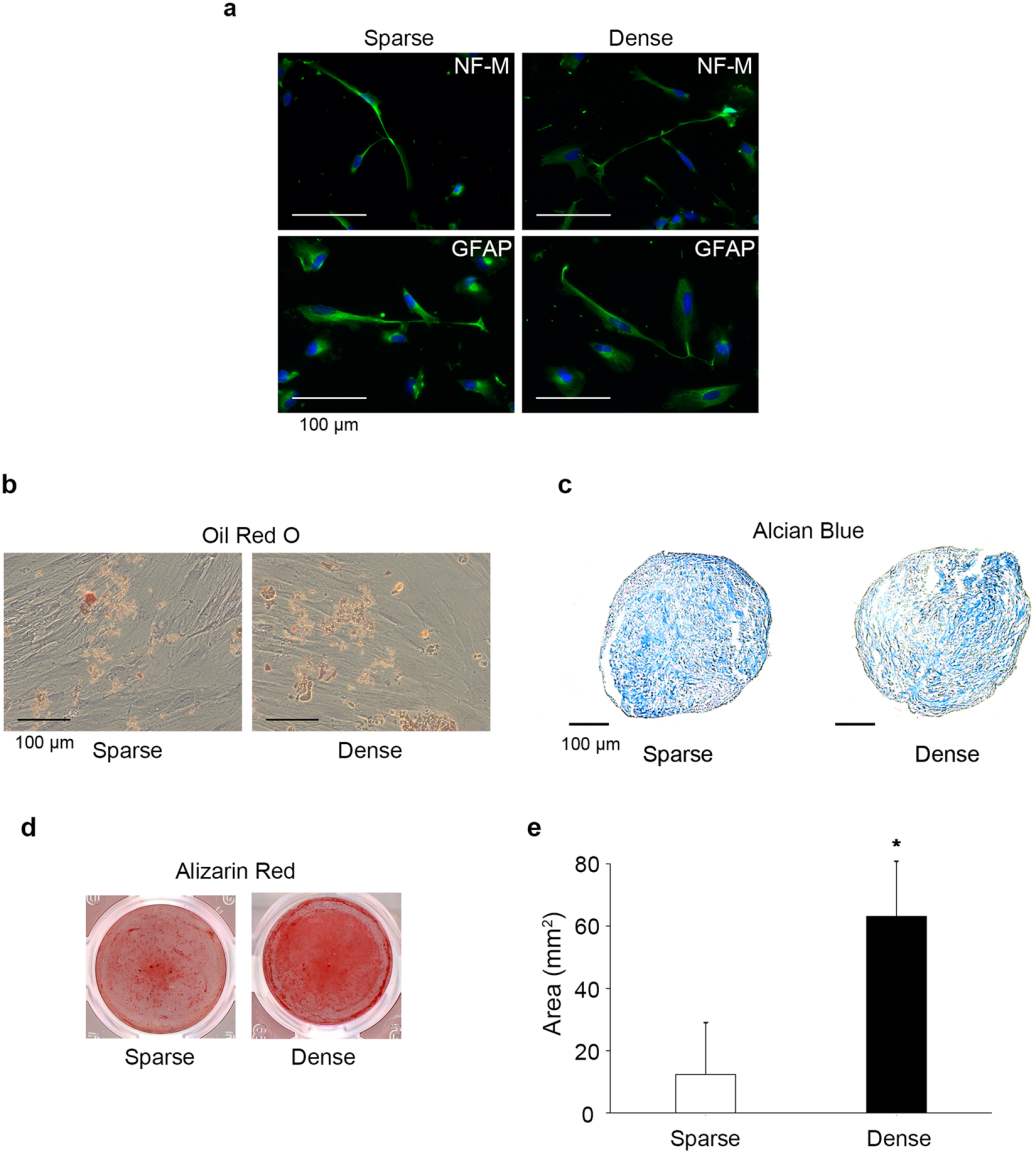
Differentiation potential. Cells in both groups were reseeded at the same cell number and cultured in differentiation medium. (**a**) Neurogenic differentiation. (**b**) Adipogenesis. (**c**) Chondrogenesis. (**d**) Osteogenesis. (**e**) Quantification of Alizarin Red-stained area. The error bar is SD (n = 4). *p = 0.019 (Mann-Whitney U test).

Next, the effects of cell culture conditions on the MSC marker expression and multi-differentiation potential of DPSCs were evaluated. Exposure to dense culture conditions for 4 days (dDPSCs) induced significant decreases of the numbers of CD73^+^ cells (92.71 ± 8.38%) and CD105^+^ cells (80.01 ± 18.40%) compared with the levels upon exposure to the sparse culture conditions for the same duration (sDPSCs) (p < 0.05; Fig. 2b). The numbers of CD44^+^ and CD90^+^ DPSCs were not altered by the dense culture conditions. Both sDPSCs and dDPSCs showed multi-differentiation potential, and neurogenic, adipogenic, chondrogenic, and osteogenic marker expression was induced by the culturing in specific differentiation medium (Fig. 3). However, mineralized nodule formation in dDPSCs was enhanced compared with that in sDPSCs (p < 0.05; Fig. 3d,e).

### Cell proliferation and *alkaline phosphatase* (*ALP)* mRNA expression

**Figure 4:**
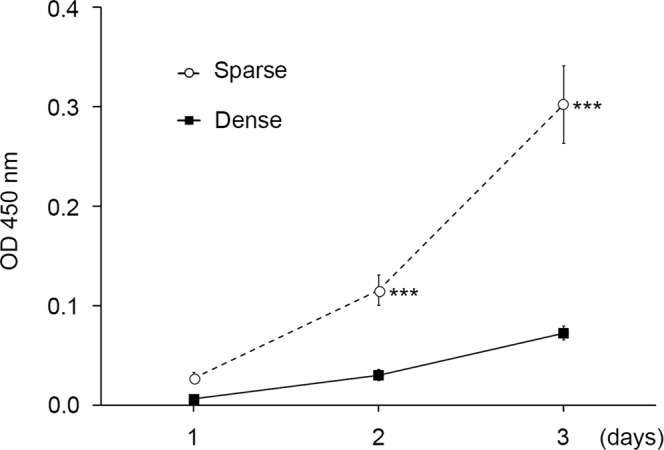
Cell proliferation. Cells in both groups were reseeded at 1 × 10^4^ cells/cm^2^ and their number was measured by the WST-8 method. The error bar is SD (n = 8). ***p = 0.0002 (Mann-Whitney U test).

**Figure 5:**
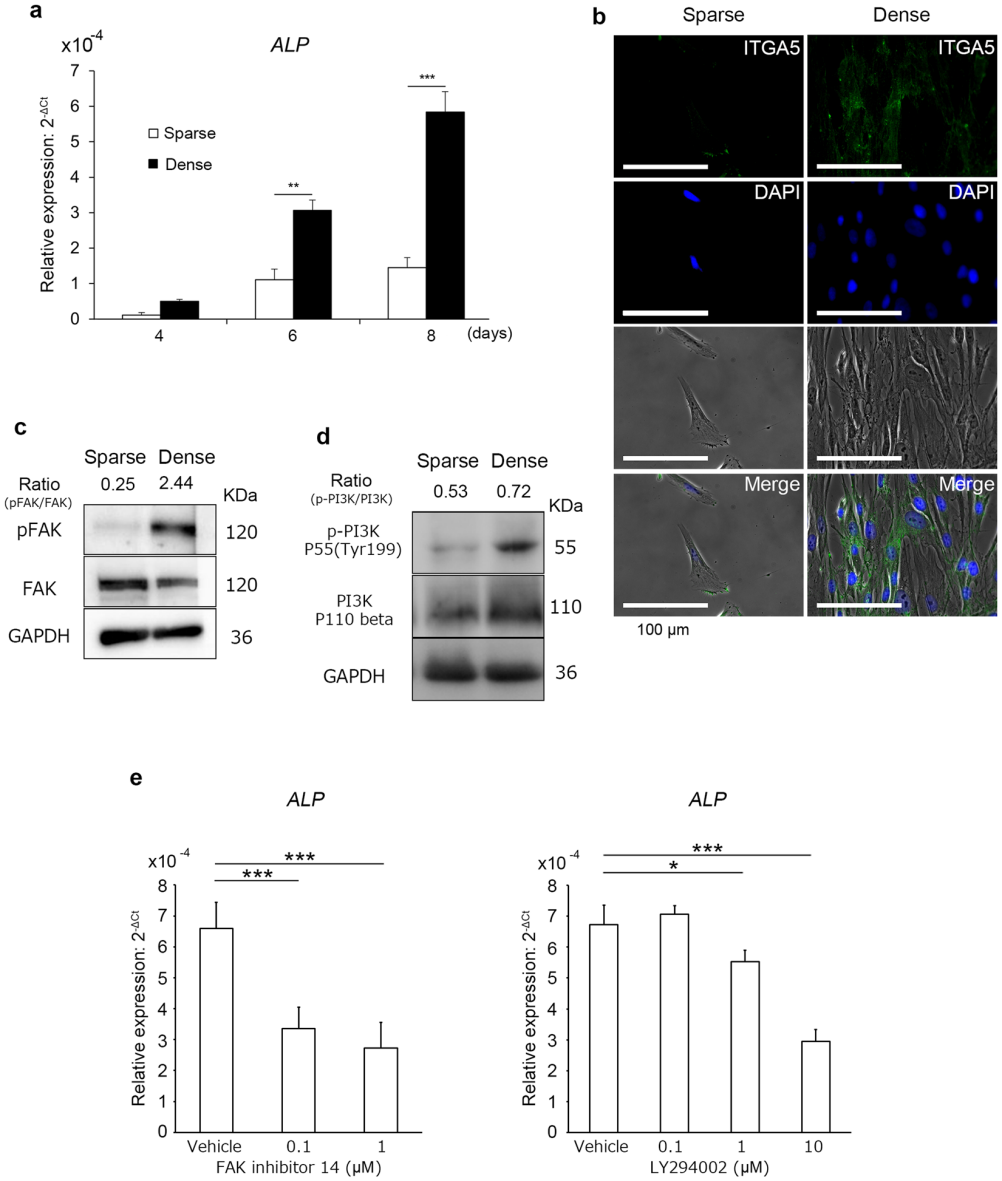
*ALP* expression and integrin, FAK, and PI3K signaling. Cells in both groups were reseeded at 1 × 10^4^ cells/cm^2^. (**a**) *ALP* mRNA expression by RT-rPCR. ΔCt was calculated as Ct(ALP) - Ct(GAPDH). The error bar is SD (n = 3). **p = 0.001 and ***p = 0.0002 (F-test and Student’s *t*-test). (**b**) Integrin alpha 5 (ITGA5) expression by immunofluorescence. (**c**) Expression of p-FAK and FAK by western blotting. (**d**) Expression of p-PI3K p55 and PI3K p110 beta by western blotting. (**e**) *ALP* mRNA expression in dDPSCs in the presence of FAK inhibitor (FAK inhibitor 14) or PI3K inhibitor (LY294002). ΔCt was calculated as Ct(ALP) - Ct(GAPDH). The error bar is SD (n = 4). *p = 0.0193 and ***p = 0.0001 (one-way ANOVA with post-hoc Dunnett’s multiple comparisons test).

The effects of the conditions for culturing DPSCs on their proliferation and expression of *ALP* mRNA, a marker of osteoblastic differentiation, were evaluated. sDPSCs and dDPSCs were seeded in equal cell density and cultured for 1 to 8 days. Cell proliferation was significantly downregulated after culturing in dense conditions (p < 0.05; Fig. 4; data from two more individual samples are shown in Supplementary Fig. S1a). The *ALP* mRNA expression of dDPSCs was significantly upregulated compared with that of sDPSCs (p < 0.05; Fig. 5a; data from two more individual samples are shown in Supplementary Fig. S1b).

The number of seeded DPSCs was varied from 1 × 10^3^ to 100 × 10^3^ cells/cm^2^ and the cells were cultured for 3 days. On the first day of cell culture, the culture at the cell density of 1 to 10 × 10^3^ cells/cm^2^ exhibited sparsely distributed cells, that at 50 × 10^3^ cells/cm^2^ was sub-confluent, and that at 100 × 10^3^ cells/cm^2^ was confluent (see Supplementary Fig. S2a). On the third day of cell culture, the culture at the cell density of 1 to 5 × 10^3^ cells/cm^2^ still had sparsely distributed cells, that at 10 × 10^3^ cells/cm^2^ was sub-confluent, and that at 50 to 100 × 10^3^ cells/cm^2^ exceeded 100% confluence (see Supplementary Fig. S2a). DPSCs seeded at 100 × 10^3^ cells/cm^2^ showed significant upregulation of *ALP* mRNA compared with DPSCs seeded at 1 × 10^3^ cells/cm^2^ (p < 0.05; see Supplementary Fig. S2b).

### Integrin/phosphoinositide 3-kinase (PI3K) signaling in densely cultured DPSCs

We next evaluated the effects of dense culture conditions on integrin signaling. Integrin alpha 5 (ITGA5) expression on dDPSCs was upregulated compared with that on sDPSC (Fig. 5b, Supplementary Fig. S3a). The expression of phosphorylated focal adhesion kinase (FAK) and phosphoinositide 3-kinase (PI3K) p55 was upregulated in dDPSCs (Fig. 5c,d), and the expression of phosphorylated PI3K p55 was promoted more in dDPSCs cultured for longer (see Supplementary Fig. S3b). Inhibitors of FAK (FAK inhibitor 14) and PI3K (LY294002) significantly downregulated the mRNA expression of *ALP* in dDPSCs (p < 0.05; Fig. 5e). FAK inhibitor 14 (except 1 µM) and LY294002 did not interfere with proliferation of DPSCs (see Supplementary Fig. S5).

### Promotion of *in vivo* mineralized-tissue formation

**Figure 6:**
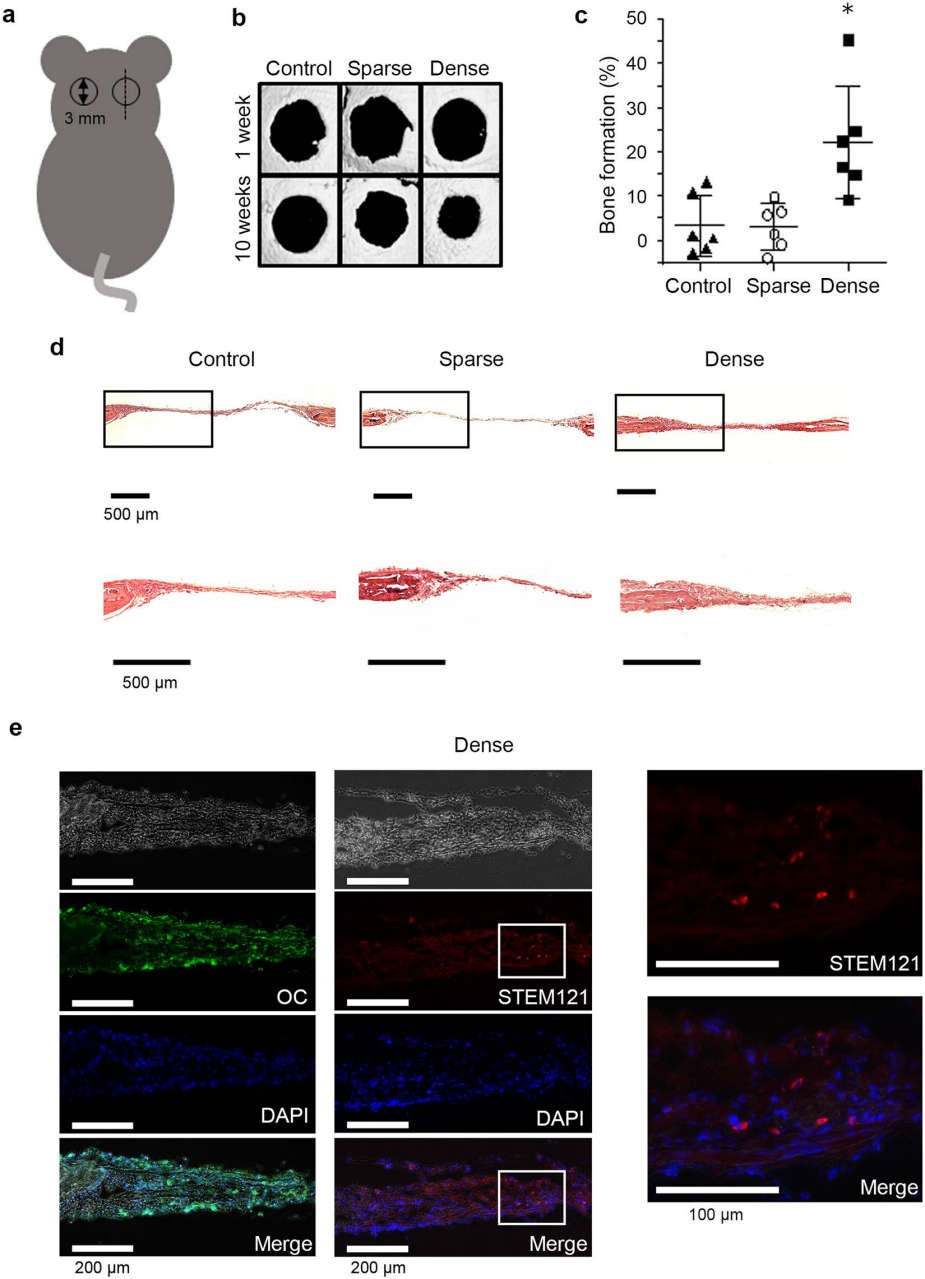
*In vivo* study. *In vivo* mineralized-tissue formation by the transplantation of DPSCs in sparse (sDPSCs) and dense (dDPSCs) groups into mouse calvarial cavities. (**a**) Schematic diagram of mouse calvarial cavities. The cavities were 3 mm in diameter and the dotted line indicates the direction of sectioning. (**b**) Micro-CT analysis. A representative figure among five cavities is shown. (**c**) Quantification of mineralized tissue formation in the bone cavities using micro-CT analysis. Bone formation (%) = 100 × (cavity area at 1 week − cavity area at 10 weeks)/(cavity area at 1 week). The error bar is SD (n = 6). *p = 0.02 (Kruskal-Wallis test with post-hoc Dunnett’s multiple comparison test). (**d**) Histological sections stained with HE. (**e**) Histological sections of the dense group immunostained with osteocalcin (OC) and human-specific antigen (STEM121).

Finally, the formation of mineralized tissue following the *in vivo* implantation of DPSCs into mouse calvarial bone cavities was evaluated. The experimental design is shown in Fig. 6a. Micro-CT analysis revealed that the transplantation of dDPSCs induced significant reduction of bone cavities compared with that of sDPSCs and control (scaffold only) (Fig. 6b,c). H-E staining revealed that a thick cell layer formed in the dDPSC-transplanted cavities (Fig. 6d). In contrast, a thin cell layer with a small number of scattered cells was formed in the sDPSC-transplanted and control cavities (Fig. 6d). Immunohistochemical staining revealed that osteocalcin was expressed in the mineralized tissue formed in the dDPSC-transplanted cavities (Fig. 6e). The transplanted dDPSCs were detected with STEM121, a human cell-recognizing antibody (Fig. 6e, Supplementary Fig. S4a,b). Radiation cytotoxicity of micro-CT was evaluated by *in vitro* cell culture system, and proliferation of DPSCs was not affected by micro-CT scanning. (see Supplementary Fig. S4c).

## Discussion

We here revealed that DPSCs cultured under dense conditions exhibited reductions of CD73^+^ and CD105^+^ populations but maintained their multi-differentiation potential and acquired higher osteogenic differentiation potential *in vitro* and elevated bone-like tissue-forming ability *in vivo*. This supported our hypothesis that cell culture density influences the differentiation pathway of DPSCs. Our findings appear to support the notion that dense culture conditions promote the generation of cells committed to osteogenic differentiation from DPSCs.

CD105, also known as endoglin, is a component of the TGF-β1 receptor complex and is commonly used as a marker of MSCs^16^. A decrease of CD105 expression has been reported in cells cultured under confluent conditions^17^, which is consistent with the present findings. Bone marrow-derived CD105^+^ MSCs are reported to be osteogenic cells^18^. However, low expression of CD105 is also reported to be linked with increased osteogenic potential^19^. The expression level of CD105 was not evaluated in dDPSCs, but the decrease of CD105^+^ cells in dDPSCs suggests that the downregulation of CD105 expression took place in a certain proportion of dDPSCs, which may have contributed to their osteogenic differentiation.

CD73, ecto-5′-nucleotidase, is also a typical MSC marker, and induces bone formation via the synthesis of adenosine, an osteoblast activator and key regulator^20,21^. The CD73 inhibitor adenosine 5′-(α,β-methylene) diphosphate induces chondrogenic matrix deposition, but reduces mineral matrix deposition and the expression of osteogenic markers^22^. Our results indicate that dDPSCs, in which the CD73^+^ population decreased, showed high osteogenicity (Figs 2b, 3d,e). CD73 expression is known to decrease with an increase in the number of passages in DPSCs^23^, while osteogenicity is preserved in cells undergoing multiple passages^24^. CD73-independent osteogenic mechanisms may also be involved in the osteogenic differentiation of dDPSCs. It has been reported that CD73 regulates other MSC markers, such as N-cadherin and vimentin, and their expression decreases following knockdown of CD73 in ovarian cancer-initiating cells^25^. However, we revealed that dDPSCs, in which CD73^+^ population decreased, showed higher N-cadherin expression compared to sDPSCs. Vimentin expression was similar between dDPSCs and sDPSCs. (see Supplementary Fig. S3b,c).

CD44 and CD90 were found to be highly expressed in dDPSCs. CD44, a transmembrane glycoprotein also referred to as P-glycoprotein 1, is a receptor for hyaluronan or hyaluronic acid^26^, and it is one of the typical markers of osteocytes^27^. CD44 is reported to regulate osteoblast differentiation negatively, and blockade of CD44 signaling with an anti-CD44 antibody induces osteoblast differentiation^28^. CD90, also known as Thy1, is a glycosylphosphatidylinositol (GPI)-anchored glycoprotein^29^, which promotes osteoblast differentiation^30^. These features indicate that signals via CD44 and CD90 may affect the osteogenic differentiation potential of dDPSCs. However, the proportions of CD44- and CD90-expressing cells were similar between sDPSCs and dDPSCs, suggesting that CD44 and CD90 make only minor contributions to the osteogenic differentiation of dDPSCs.

One of the major properties of MSCs is their multi-differentiation potential, and both dDPSCs and sDPSCs possess neurogenic, adipogenic, chondrogenic, and osteogenic differentiation capacity (Fig. 3). Sparse culture conditions have been reported to be essential for maintenance of the multi-differentiation potential of BMSCs^12^, but we revealed that dense culture for 4 days did not reduce the multi-differentiation potential of DPSCs (Fig. 3). Knockdown of CD90 is reported to induce a shift in the stemness of MSCs towards a state that is more susceptible to differentiation^29^. However, most dDPSCs expressed CD90, and thus the maintenance of CD90 expression may be related to preservation of the multi-differentiation potential of dDPSCs. However, the mechanisms behind the maintenance or loss of stemness and multi-differentiation potential still require further investigation.

dDPSCs showed high osteogenic differentiation potential compared with sDPSCs (Figs 3d,e and 6). Cell-cell interaction and cell density play important roles in osteogenesis in some cell-lines (BMSCs^31^, mouse MCSs^32^ and MC3T3-E1^33^). A dense cell population upon culture induces tight cell–cell contacts, which may further activate cell contact-dependent signaling such as integrin and cadherin signalings. Integrins are transmembrane receptors and adhesion molecules that mediate various intracellular events involving cell differentiation^34^. ITGA5 is involved in osteoblastic differentiation and osteogenesis, and activation of endogenous ITGA5 by application of dexamethasone promotes osteoblastic/osteogenic differentiation via PI3K signaling in BMSCs^35^. We have reported that conditions involving high cell–cell contact induced by culturing in spheroid conditions using non-adherent culture dish caused the upregulation of phosphorylated FAK and paxillin expression in mouse dental papilla cells^36^. We revealed that expression of ITGA5 (Fig. 5b), N-cadherin (see Supplementary Fig. S3c), phosphorylated FAK (Fig. 5c) and phosphorylated PI3K (Fig. 5d) was upregulated in dDPSCs. Moreover, FAK or PI3K inhibitors significantly downregulated the expression of *ALP* mRNA in dDPSCs (Fig. 5e) independent of cell proliferation^37^ except a high concentration of the FAK inhibitor (see Supplementary Fig. S5). These findings may support the notion that FAK/PI3K signaling, a major stream of the integrin signaling cascade in osteoblast differentiation^34,38–40^, is involved in the commitment of dDPSCs toward osteogenic differentiation. Cadherin signaling is another essential signaling in cell-cell interaction, and crosstalk between integrin and cadherin induces activation of FAK/PI3K signaling^34,41^. Increased expression of ITGA5 (Fig. 5b) and N-cadherin (see Supplementary Fig. S3c) in dDPSCs suggests that both signaling pathways may induce their osteogenic differentiation independently or synergically.

The proliferation of dDPSCs was lower than that of sDPSCs, which agrees with the finding that the proliferation of BMSCs is downregulated upon seeding at a high cell density^11,13^. In contrast to trypsinization and low-density seeding that stimulate cell proliferation^42^, high-density seeding and continuous tight cell contact most probably induce the “inhibition of contacts” in which cell modifications and depressed cell proliferation capacity occur^11,13,43^. Furthermore, the commitment of dDPSCs to osteogenic differentiation may affect their capacity to proliferate, since terminal differentiation can lead to exit from the cell cycle^44^.

The *in vivo* implantation of dDPSCs into mouse calvarial bone cavities resulted in the enhanced formation of bone-like tissues compared with that of sDPSCs (Fig. 6). *In vivo* mineralized tissue formation following the transplantation of MSCs has been reported, but the pre-culture of MSCs in osteogenic differentiation medium is recommended before application^45^. In this paper, we present a new approach to induce the osteogenic commitment of DPSCs by simply culturing them under dense conditions without using a specific medium for osteogenic commitment. Xenotransplantation of three-dimensional cultured human BMSCs/extracellular matrix complex in mouse calvarial defects induces bone-like tissue formation^46^ similar to our results. However, further study is necessary to determine whether the promoted osteogenic capacity is a common property of MSCs or specific to BMSCs/DPSCs.

It is also still unclear whether transplanted DPSCs or recruited host stem cells are more responsible for the bone tissue regeneration. Transplanted MSCs secrete homing factors, which induce the recruitment of host stem cells, and infiltrated host stem cells are involved in the regeneration of tissues^47,48^. However, regenerated bone tissue expressed human-specific antigen (STEM121) (Fig. 6e, Supplementary Fig. S4a,b), indicating that transplanted DPSCs had differentiated into osteoblasts at the transplanted area, as previously reported^49^.

In summary, our findings showed that dense culture induced the downregulation of CD73^+^ and CD105^+^ populations in DPSCs, but there was no difference concerning the multi-differentiation potential between dDPSCs and sDPSCs. Osteogenic differentiation was induced in dDPSCs, featuring upregulation of the expression of phosphorylated FAK and PI3K, which are downstream of integrin signaling. Moreover, mineralized tissue formation was promoted by the transplantation of dDPSCs into mouse calvarial bone cavities *in vivo*. From these findings, it can be concluded that dense culture conditions modified the properties of DPSCs and gave rise to cells more committed to osteogenic differentiation via integrin signaling. The results also suggest that dense culture conditions favor the propagation of DPSCs to be used for mineralized tissue regeneration.

## Materials and Methods

### Isolation of dental pulp stem cells (DPSCs)

Freshly extracted wisdom teeth (n = 7; 20 to 33 years old; approved by the Ethical Committee of Tokyo Medical and Dental University, #D2014-039-01) were kept in chilled Hanks’ balanced salt solution supplemented with amphotericin B (2.5 mg/ml; Wako Pure Chemical Industries, Osaka, Japan) and gentamycin (3 mg/ml, Wako Pure Chemical Industries) for 1 to 12 h until the experiments. Guide grooves were prepared on the buccal, occlusal, and lingual aspects of the crown and root of extracted teeth with diamond bars (#AR2; GC Corporation, Tokyo, Japan), avoiding pulp exposure, and the grooved teeth were easily divided into two pieces with a chisel and a mallet. All the pulp tissues extracted from the divided tooth pieces were minced and digested with Liberase (Roche, Basel, Switzerland) for 40 min at 37 °C and a single-cell suspension was obtained with a cell strainer (70 µm diameter; BD Falcon, Franklin Lakes, NJ, USA). Then, cells were seeded into 100-mm cell culture dishes (CellBIND Surface, Corning, NY, USA) in sparse conditions, and were cultured in alpha-modified minimum essential medium (α-MEM; Wako Pure Chemical Industries) containing 10% fetal bovine serum (FBS; Thermo Fisher Scientific, Waltham, MA, USA) and an antibiotic and antifungal solution (penicillin–streptomycin–amphotericin B suspension; Wako Pure Chemical Industries) at 37 °C and 5% CO_2_ for 7–10 days to generate cell colonies. The colony-forming cells, which were regarded as DPSCs, were collected using 0.05% trypsin solution and expanded under sparse conditions (5 × 10^3^ to 1 × 10^4^ cells/cm^2^) for three to six passages to obtain a sufficient number of cells for the experiments. The experimental scheme is shown in Fig. 1.

### Modification of cell culture conditions

DPSCs were seeded in sparse (sDPSCs; 5 × 10^3^ cells/cm^2^) or dense (dDPSCs; 1 × 10^5^ cells/cm^2^) conditions and cultured for 4 days. sDPSCs were passaged at 3 days to maintain their sparsity, whereas the culture medium was changed only at 3 days for dDPSCs. Following 4 days of culture, DPSCs in each group were collected by trypsinization, and the same number of cells in each group was used for further experiments.

### Flow cytometry

DPSCs (5 × 10^5^ cells) were washed with FACS buffer [phosphate-buffered saline (PBS) containing 2% FBS] and then incubated with FITC mouse IgG1κ isotype control antibody (1:100, 1:200, MOPC-21; BioLegend, San Diego, CA, USA), FITC-labeled anti-CD34 (1:200, 4H11; eBioscience/Affymetrix, Santa Clara, CA, USA), anti-CD44 (1:100, Bu52; Serotec/Bio-Rad, Hercules, CA, USA), anti-CD73 (1:200, AD2; eBioscience/Affymetrix), anti-CD90 (1:100, F15-42-1; Serotec/Bio-Rad), anti-CD105 (1:100, SN6; Serotec/Bio-Rad), and anti-CD146 (1:100, OJ79c; Serotec/Bio-Rad) for 20 min on ice. Next, the DPSCs were washed twice with FACS buffer and applied to a flow cytometer (FACS Calibur; BD Biosciences, San Jose, CA, USA).

### Cell proliferation assay

The proliferation of DPSCs was measured at 1, 2, and 3 days with Cell Counting Kit-8 (Dojindo Laboratories, Kumamoto, Japan) following the manufacturer’s instructions. Briefly, cells were seeded in 96-well plates (3 × 10^3^ cells/100 µl medium/well) and incubated with WST-8 solution. The absorbance was measured at 450 nm using a microplate reader (Tecan, Männedorf, Switzerland).

### Neurogenic differentiation

DPSCs were seeded on fibronectin (Wako Pure Chemical Industries)-coated 8-well cell culture slides (SPL Life Sciences, Gyeonggi-do, South Korea), and were cultured in a neurogenic differentiation medium containing B-27 supplement (Gibco BRL, Palo Alto, CA, USA) for 3 days. Following fixation with 4% paraformaldehyde (PFA) for 30 min at room temperature, neurogenic differentiation was evaluated by the expression of neuronal markers, neurofilament medium (NF-M) and glia fibrillary acidic protein (GFAP), which were detected by immunohistochemical staining using anti-NF-M and anti-GFAP antibodies. The staining protocol is described in the section entitled “Immunohistochemistry.”

### Adipogenic differentiation

DPSCs (6 × 10^3^ cells) were seeded on cell disks (2 cm^2^; Sumitomo Bakelite, Tokyo, Japan) and cultured in an adipogenic differentiation medium containing methylisobutylxanthine (0.5 mM; Wako Pure Chemical Industries), dexamethasone (1 µM; Wako Pure Chemical Industries), and recombinant human insulin (10 µg/ml; Wako Pure Chemical Industries) for 25 days. Cells were fixed with 4% PFA for 30 min at room temperature and Oil Red O (Sigma, St. Louis, MO, USA) staining was performed.

### Chondrogenic differentiation

DPSCs (1 × 10^3^ cells) were seeded in 96-well low-attachment culture plates (PrimeSurface; Sumitomo Bakelite) and cultured in a chondrogenic differentiation medium (Mesenchymal Stem Cell Chondrogenic Differentiation Medium; PromoCell, Heidelberg, Germany) for 25 days. Pellets were fixed in 4% PFA for 24 h at 4 °C and embedded in OCT compound (Sakura Finetek Co., Ltd., Tokyo, Japan). Frozen sections (5 µm thickness) were prepared and stained with Alcian Blue (Wako Pure Chemical Industries).

### Osteogenic differentiation

DPSCs (2 × 10^5^ cells) were seeded in 48-well cell culture plates and cultured in an osteogenic differentiation medium containing L-ascorbic acid-2-phosphate (0.2 mM; Wako Pure Chemical Industries), beta-glycerophosphate (5 mM; Wako Pure Chemical Industries), dexamethasone (1 nM; Wako Pure Chemical Industries), and bone morphogenic protein-2 (BMP-2, 100 ng/ml; Wako Pure Chemical Industries) for 25 days. Calcified nodules were stained with Alizarin Red S (Wako Pure Chemical Industries) after methanol fixation for 1 min at room temperature. The volume of mineralized nodules stained with Alizarine Red S was measured by ImageJ software (https://imagej.nih.gov/ij/).

### RNA isolation and real-time PCR

Total RNAs were isolated with QuickGene-Mini80 (Kurabo, Tokyo, Japan) and cDNA was synthesized with PrimeScript™ RT Master Mix (Takara Bio Inc., Kusatsu, Japan). Real-time RT-PCR was performed with specific primers for alkaline phosphatase (ALP, forward: 5′-ATGCTGAGTGACACAGACAAGAAG-3′, reverse: 5′-GGTAGTTGTTGTGAGCATAGTCCAC-3′, 124 bp), glyceraldehyde 3-phosphate dehydrogenase (GAPDH, forward: 5′-GGCCTCCAAGGAGTAAGACC-3′, reverse: 5′-AGGGGTCTACATGGCAACTG-3′, 147 bp), and Go Taq qPCR Master Mix (Promega, Madison, WI, USA) using the CFX96 qPCR system (Bio-Rad). ΔCt was calculated as Ct(ALP) - Ct(GAPDH). Y-axis in Figs 5a,e, S1b, S2b is relative expression: 2^−ΔCt^.

### Western blotting

DPSCs (5 × 10^5^ cells) were lysed in 100 µl of RIPA buffer (25 mM Tris/HCl pH 7.4, 150 mM NaCl, 10 mM MgCl_2_, 1 mM EDTA, 1% NP-40, 5% glycerol) containing PhosSTOP EASY pack (Roche) and cOmplete protease inhibitor cocktail (Roche). Samples were separated with 10% SDS-PAGE and transferred to Immobilon transfer membranes (Merck Millipore, Darmstadt, Germany). After blocking with PVDF blocking reagent (Toyobo, Osaka, Japan), the membrane was incubated with anti-FAK antibody (1:500; Signalway Antibody, College Park, MD, USA), anti-phospho-FAK (Tyr397) antibody (1:500; Cell Signaling Technology), anti-PI3K p110 beta antibody (1:1000; Cell Signaling Technology, Danvers, MA, USA), anti-phospho-PI3K p55 (Tyr199) antibody (1:1000; Cell Signaling Technology), anti-N-cadherin antibody (1:400; BD Biosciences), anti-vimentin antibody (no dilution, a ready-to-use antibody; Dako/Agilent, Santa Clara, CA, USA) or HRP-labeled anti-GAPDH antibody (1:10,000; MBL, Nagoya, Japan) overnight at 4 °C. After washing with Tris-buffered saline containing Tween 20 (1% v/v), the membrane was incubated with peroxidase-conjugated anti-rabbit IgG (1:5000; Jackson ImmunoResearch Labs, West Grove, PA, USA) for 1 h at room temperature, except for HRP-labeled anti-GAPDH antibody-applied membranes. Corresponding bands were visualized using Luminata Forte (Merck Millipore), captured by the digital imaging system LAS 3000 Mini (Fujifilm Tokyo, Japan), and measured by ImageJ software.

### Inhibitors

FAK inhibitor (FAK inhibitor 14; Cayman Chemical, Ann Arbor, MI, USA) and PI3K inhibitor (LY294002; Cayman Chemical) were dissolved in dimethyl sulfoxide (Sigma) and used at appropriate concentrations.

### Transplantation of DPSCs into experimentally created mouse calvarial bone cavities

All animal experiments were approved by the Institutional Animal Care and Use Committee of Tokyo Medical and Dental University (A2017-155A). Six-week-old male C57BL/6JJcl mice were obtained from CLEA Japan (Tokyo, Japan). Animals were anesthetized by the intraperitoneal injection of a mixture of ketamine hydrochloride (50 mg/kg, Ketalar; Sankyo, Tokyo, Japan) and xylazine hydrochloride (10 mg/kg, Selactar; Bayer Yakuhin, Osaka, Japan). A sagittal incision was made on the scalp and the calvarium was exposed. The outline of the bone cavity was marked with a biopsy punch (diameter 3 mm; Kai Medical, Gifu, Japan), and the calvarial bone was removed with an ultrasonic scaling device (Solfy F; Morita, Tokyo, Japan) under cool saline irrigation. Pellets of sDPSCs and dDPSCs (3 × 10^5^ cells each) were inserted into a cooled native collagen gel scaffold (15 µl, Cellgen I-AC; Koken, Tokyo, Japan. The gels were prepared according to the manufacturer’s protocol for 3D culture: 80% v/v diluted using α-MEM, HEPES pH 7.4, NaHCO_3_ and water), and a collagen scaffold containing cells was carefully placed on the bone cavities (n = 6 per group). A collagen scaffold without cells was used as a control (n = 6). Then, the skin was sutured with 6-0 nylon. Micro-CT scans (80 kV, 30 µA; inspeXio SMX-100CT; Shimadzu, Kyoto, Japan) were taken at 1 and 10 weeks after surgery under anesthesia (same formula as for the surgery experiment). The image slices were reconstructed using VGStudio MAX software (Volume Graphics, Aichi, Japan) and measured by ImageJ software. The animals were sacrificed at 10 weeks after surgery under carbon dioxide euthanasia. The calvaria were carefully extracted and fixed with 4% PFA for 24 h at 4 °C and demineralized with 0.5 M EDTA for 3 weeks at 4 °C. Samples were embedded in OCT compound and cryostat sections (5 µm) were prepared for histological evaluation. To evaluate the cytotoxicity of radiation, *in vitro* cultured DPSCs were scanned by micro-CT (80 kV, 30 µA; inspeXio SMX-100CT), and their proliferation was compared to non-scanned DPSCs.

### Immunohistochemistry

Sections were incubated with 10% normal serum (horse for monoclonal antibodies, goat for polyclonal antibodies) for 10 min at room temperature to prevent the nonspecific binding of antibodies. Anti-GFAP rabbit polyclonal antibody (1:500; GeneTex, Irvine, CA, USA), anti-NF-M mouse monoclonal antibody (1:200; GeneTex), anti-ITGA5 mouse monoclonal antibody (1:1000; Abcam, Cambridge, UK), anti-osteocalcin rabbit polyclonal antibody (1:500; BioSS Biological Technology, Beijing, China), and STEM121 (mouse monoclonal antibody specific for human cytoplasmic marker, 1:1000; Takara Bio Inc.) were used for primary antibodies, and samples were incubated with one of the primary antibodies overnight at 4 °C. Alexa Fluor 488-conjugated goat anti-rabbit or -mouse IgG (1:500; Molecular Probes, Eugene, OR, USA) or Alexa Fluor 594-conjugated goat anti-rabbit IgG (1:500; Molecular Probes) was used as a secondary antibody and applied for 30 min at room temperature. DAPI (NucBlue Fixed Cell Ready Probes Reagent; Thermo Fisher) was used for nuclear staining. Positively stained areas were measured by ImageJ software.

### Statistical analysis

Statistical analysis was performed using one-way ANOVA, Kruskal-Wallis test, Dunn’s multiple comparison test, F-test, Mann-Whitney U test and Student’s *t*-test. *P* values less than 0.05 were considered statistically significant.

## Supplementary information

Figure S1, S2, S3, S4, S5

## Data Availability

The datasets used and/or analyzed during the current study are available from the corresponding author on reasonable request.

## References

[1] Kawashima N (2012). Characterisation of dental pulp stem cells: A new horizon for tissue regeneration?. Arch. Oral Biol..

[2] Squillaro T, Peluso G, Galderisi U (2016). Clinical trials with mesenchymal stem cells: an update. Cell Transplant..

[3] Gronthos S, Mankani M, Brahim J, Robey PG, Shi S (2000). Postnatal human dental pulp stem cells (DPSCs) *in vitro* and *in vivo*. Proc. Natl. Acad. Sci. USA.

[4] Iohara K (2006). Side population cells isolated from porcine dental pulp tissue with self-renewal and multipotency for dentinogenesis, chondrogenesis, adipogenesis, and neurogenesis. Stem Cells.

[5] Jensen J (2016). Dental pulp-derived stromal cells exhibit a higher osteogenic potency than bone marrow-derived stromal cells *in vitro* and in a porcine critical-size bone defect model. Sicot-J..

[6] D’Aquino R (2009). Human mandible bone defect repair by the grafting of dental pulp stem/progenitor cells and collagen sponge biocomplexes. Eur. Cells Mater..

[7] Nakashima M, Iohara K (2014). Mobilized dental pulp stem cells for pulp regeneration: Initiation of clinical trial. J. Endod..

[8] Huang GT-J, Gronthos S, Shi S (2009). Mesenchymal stem cells derived from dental tissues vs.those from other sources: their biology and role in regenerative medicine. J. Dent. Res..

[9] Miura M (2003). SHED: Stem cells from human exfoliated deciduous teeth. Proc. Natl. Acad. Sci..

[10] Kawashima N, Noda S, Yamamoto M, Okiji T (2017). Properties of dental pulp–derived mesenchymal stem cells and the effects of culture conditions. J. Endod..

[11] Sotiropoulou PA, Perez SA, Salagianni M, Baxevanis CN, Papamichail M (2006). Characterization of the optimal culture conditions for clinical scale production of human mesenchymal stem cells. Stem Cells.

[12] Sekiya I (2002). Expansion of human adult stem cells from bone marrow stroma: conditions that maximize the yields of early progenitors and evaluate their quality. Stem Cells.

[13] Abo-Aziza FA-AM, Zaki AE-KA (2016). The impact of confluence on BMMSC proliferation and osteogenic differentiation&quot;. Int. J. Hematol. Stem Cell Res..

[14] Suchánek J (2007). Human dental pulp stem cells – isolation and long term cultivation. Acta. Medica. Cordoba..

[15] Lizier NF (2012). Scaling-up of dental pulp stem cells isolated from multiple niches. PLoS One.

[16] Dominici M (2006). Minimal criteria for defining multipotent mesenchymal stromal cells. The International Society for Cellular Therapy position statement. Cytotherapy.

[17] Anderson P, Carrillo-Gálvez AB, García-Pérez A, Cobo M, Martín F (2013). CD105 (endoglin)-negative murine mesenchymal stromal cells define a new multipotent subpopulation with distinct differentiation and immunomodulatory capacities. PLoS One.

[18] Aslan H (2006). Osteogenic differentiation of noncultured immunoisolated bone marrow-derived CD105 + cells. Stem Cells.

[19] Levi B (2011). CD105 protein depletion enhances human adipose-derived stromal cell osteogenesis through reduction of transforming growth factor β1 (TGF-β1) signaling. J. Biol. Chem..

[20] Bradaschia-Correa V (2017). Ecto-5′-nucleotidase (CD73) regulates bone formation and remodeling during intramembranous bone repair in aging mice. Tissue Cell.

[21] Takedachi M (2012). CD73-generated adenosine promotes osteoblast differentiation. J. Cell. Physiol..

[22] Ode A (2012). CD73/5′-ecto-nucleotidase acts as a regulatory factor in osteo-/chondrogenic differentiation of mechanically stimulated mesenchymal stromal cells. Eur. Cells Mater..

[23] Sivasankar V, Ranganathan K (2016). Growth characteristics and expression of CD73 and CD146 in cells cultured from dental pulp. J. Investig. Clin. Dent..

[24] Yu J (2010). Differentiation potential of STRO-1+ dental pulp stem cells changes during cell passaging. BMC Cell Biol..

[25] Lupia M (2018). CD73 regulates stemness and epithelial-mesenchymal transition in ovarian cancer-initiating cells. Stem Cell Reports.

[26] Senbanjo LT, Chellaiah MA (2017). CD44: A multifunctional cell surface adhesion receptor is a regulator of progression and metastasis of cancer cells. Front. Cell Dev. Biol..

[27] Hughes DE, Salter DM, Simpson R (1994). CD44 expression in human bone: A novel marker of osteocytic differentiation. J. Bone Miner. Res..

[28] Kaneko K (2015). Hyaluronan inhibits BMP-induced osteoblast differentiation. FEBS Lett..

[29] Moraes DA (2016). A reduction in CD90 (THY-1) expression results in increased differentiation of mesenchymal stromal cells. Stem Cell Res. Ther..

[30] Paine A (2018). Thy1 is a positive regulator of osteoblast differentiation and modulates bone homeostasis in obese mice. FASEB J..

[31] Eyckmans J, Lin GL, Chen CS (2012). Adhesive and mechanical regulation of mesenchymal stem cell differentiation in human bone marrow and periosteum-derived progenitor cells. Biol. Open..

[32] Mao AS, Shin JW, Mooney DJ (2016). Effects of substrate stiffness and cell-cell contact on mesenchymal stem cell differentiation. Biomaterials.

[33] Guntur AR, Rosen CJ, Naski MC (2012). N-cadherin adherens junctions mediate osteogenesis through PI3K signaling. Bone.

[34] Marie PJ, Haÿ E, Saidak Z (2014). Integrin and cadherin signaling in bone: Role and potential therapeutic targets. Trends Endocrinol. Metab..

[35] Hamidouche Z (2009). Priming integrin alpha5 promotes human mesenchymal stromal cell osteoblast differentiation and osteogenesis. Proc. Natl. Acad. Sci. USA.

[36] Yamamoto M (2014). Three-dimensional spheroid culture promotes odonto/osteoblastic differentiation of dental pulp cells. Arch. Oral Biol..

[37] Gilmore AP, Romer LH (1996). Inhibition of focal adhesion kinase (FAK) signaling in focal adhesions decreases cell motility and proliferation. Mol. Biol. Cell.

[38] Salasznyk RM, Klees RF, Williams WA, Boskey A, Plopper GE (2007). Focal adhesion kinase signaling pathways regulate the osteogenic differentiation of human mesenchymal stem cells. Exp. Cell Res..

[39] McGonnell IM, Grigoriadis AE, Lam EWF, Price JS, Sunters A (2012). A specific role for phosphoinositide 3-kinase and AKT in osteoblasts?. Front. Endocrinol. (Lausanne)..

[40] Baker N, Sohn J, Tuan RS (2015). Promotion of human mesenchymal stem cell osteogenesis by PI3-kinase/Akt signaling, and the influence of caveolin-1/cholesterol homeostasis. Stem Cell Res. Ther..

[41] Mui KL, Chen CS, Assoian RK (2016). The mechanical regulation of integrin-cadherin crosstalk organizes cells, signaling and forces. J. Cell Sci..

[42] Maizel A, Nicolini C, Baserga R (1975). Effect of cell trypsinization on nuclear proteins of WI-38 fibroblasts in culture. J. Cell. Physiol..

[43] McClatchey AI, Yap AS (2012). Contact inhibition (of proliferation) redux. Curr. Opin. Cell Biol..

[44] Buttitta LA, Edgar BA (2007). Mechanisms controlling cell cycle exit upon terminal differentiation. Curr. Opin. Cell Biol..

[45] Morad G, Kheiri L, Khojasteh A (2013). Dental pulp stem cells for *in vivo* bone regeneration: A systematic review of literature. Arch. Oral Biol..

[46] Takeshita K (2017). Xenotransplantation of interferon-gamma-pretreated clumps of a human mesenchymal stem cell/extracellular matrix complex induces mouse calvarial bone regeneration. Stem Cell Res. Ther..

[47] Gnecchi M (2005). Paracrine action accounts for marked protection of ischemic heart by Akt-modified mesenchymal stem cells [2]. Nat. Med..

[48] Inukai T (2013). Novel application of stem cell-derived factors for periodontal regeneration. Biochem. Biophys. Res. Commun..

[49] Gronthos S (2002). Stem cell properties of human dental pulp stem cells. J. Dent. Res..

