# Supplementary material for: Effect of cell culture density on dental pulp-derived mesenchymal stem cells with reference to osteogenic differentiation
Source: Sci Rep. 2019 Apr 1;9:5430. doi: 10.1038/s41598-019-41741-w (PMC6443725; doi:10.1038/s41598-019-41741-w)
Supplement: Supplementary file 1 — Figure S1, S2, S3, S4, S5 [file 41598_2019_41741_MOESM1_ESM.pdf]

Effect of cell culture density on dental pulp-derived mesenchymal stem cells with reference to osteogenic differentiation

Sonoko Noda, Nobuyuki Kawashima, Mioko Yamamoto,
Kentaro Hashimoto, Keisuke Nara, Ichiro Sekiya, Takashi Okiji

Supplementary Figure.S1~5

Fig. S1

a

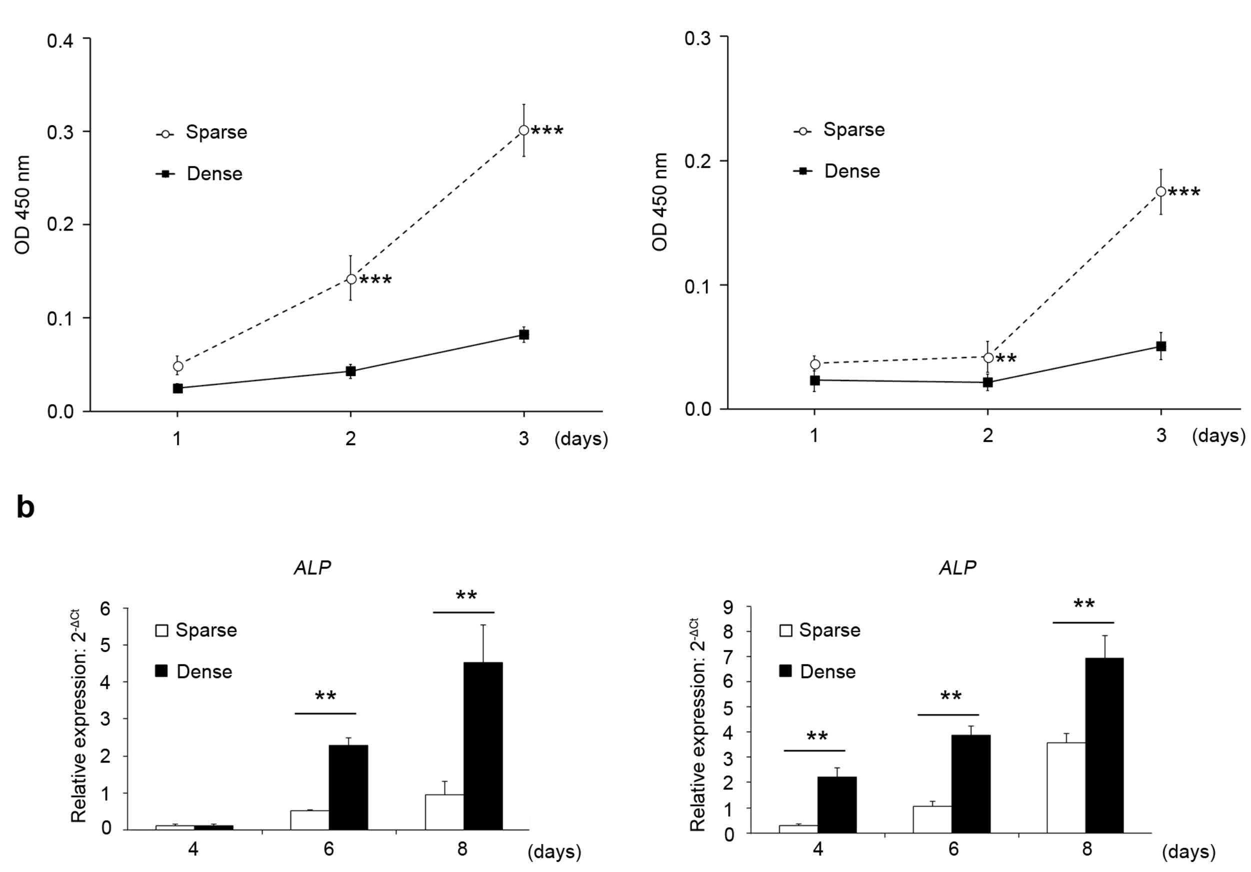

Figure S1. Cell proliferation and *ALP* mRNA expression of DPSCs in sparse (sDPSCs) and dense (dDPSCs) groups. (a) Cell proliferation. The error bar is SD (n = 7, 2 donors). ***: p = 0.0006 and **: p = 0.0012 (Mann-Whitney U test). (b) *ALP* mRNA expression. The error bar is SD (n = 3, 2 donors). ΔCt was calculated as Ct (ALP) - Ct (GAPDH). **: p = 0.004 (F-test and Student's *t*-test).

Fig. S2

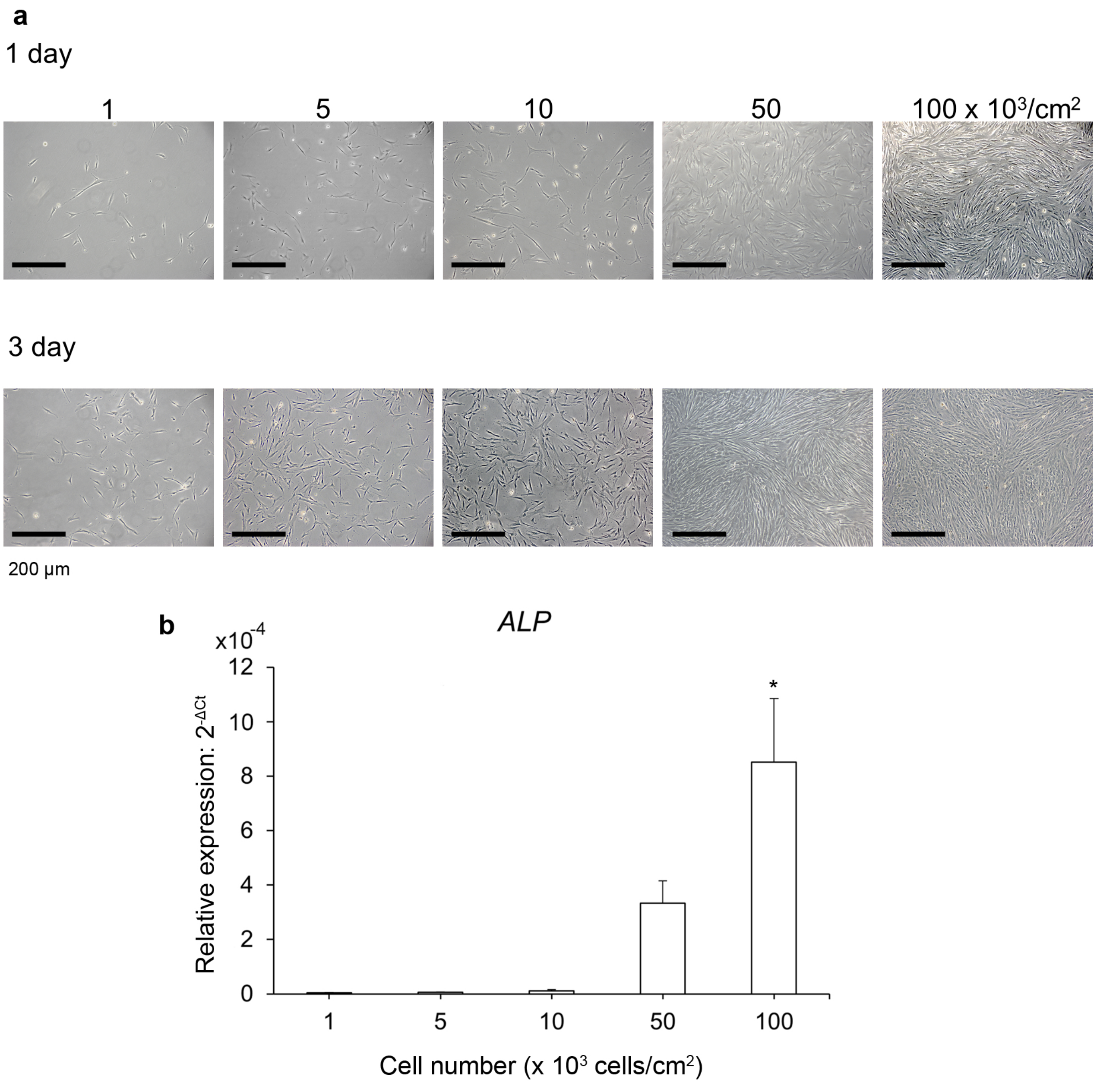

Figure S2. Phase contact photographs and *ALP* mRNA expression of DPSCs in different seeding densities. (a) Phase contrast photographs of DPSCs at 1 and 3 days after seeding concentration (1, 5, 10, 50 and 100 x 10^3 cells/cm^2). (b) *ALP* mRNA expression of DPSCs was evaluated at 3 days. ΔCt was calculated as Ct (ALP) - Ct (GAPDH). The error bar is SD (n=4). *: p = 0.0202 (Kruskal–Wallis test with post-hoc Dunnett's multiple comparison test).

Fig. S3

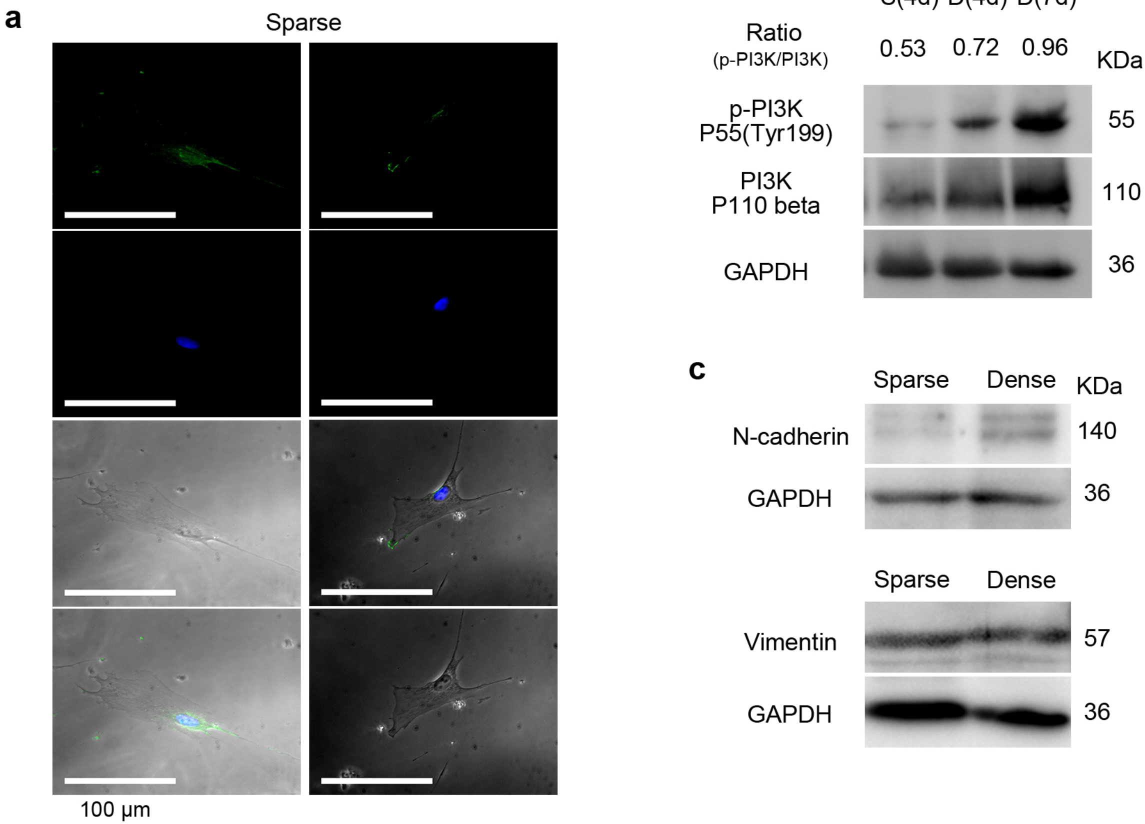

Figure S3. Integrin alpha 5 (ITGA5) expression of DPSCs by immunohistochemistry and PI3K, N-cadherin and vimentin expression of DPSCs by western blotting. (a) Integrin alpha 5 (ITGA5) expression in sDPSCs. (b) Expression of p-PI3K p55 and PI3K p110 beta in dense (dDPSCs) group cultured for 7 days (7d). (c) Expression of N-cadherin and vimentin in sparse (sDPSCs) and dense (dDPSCs) groups.

Fig. S4

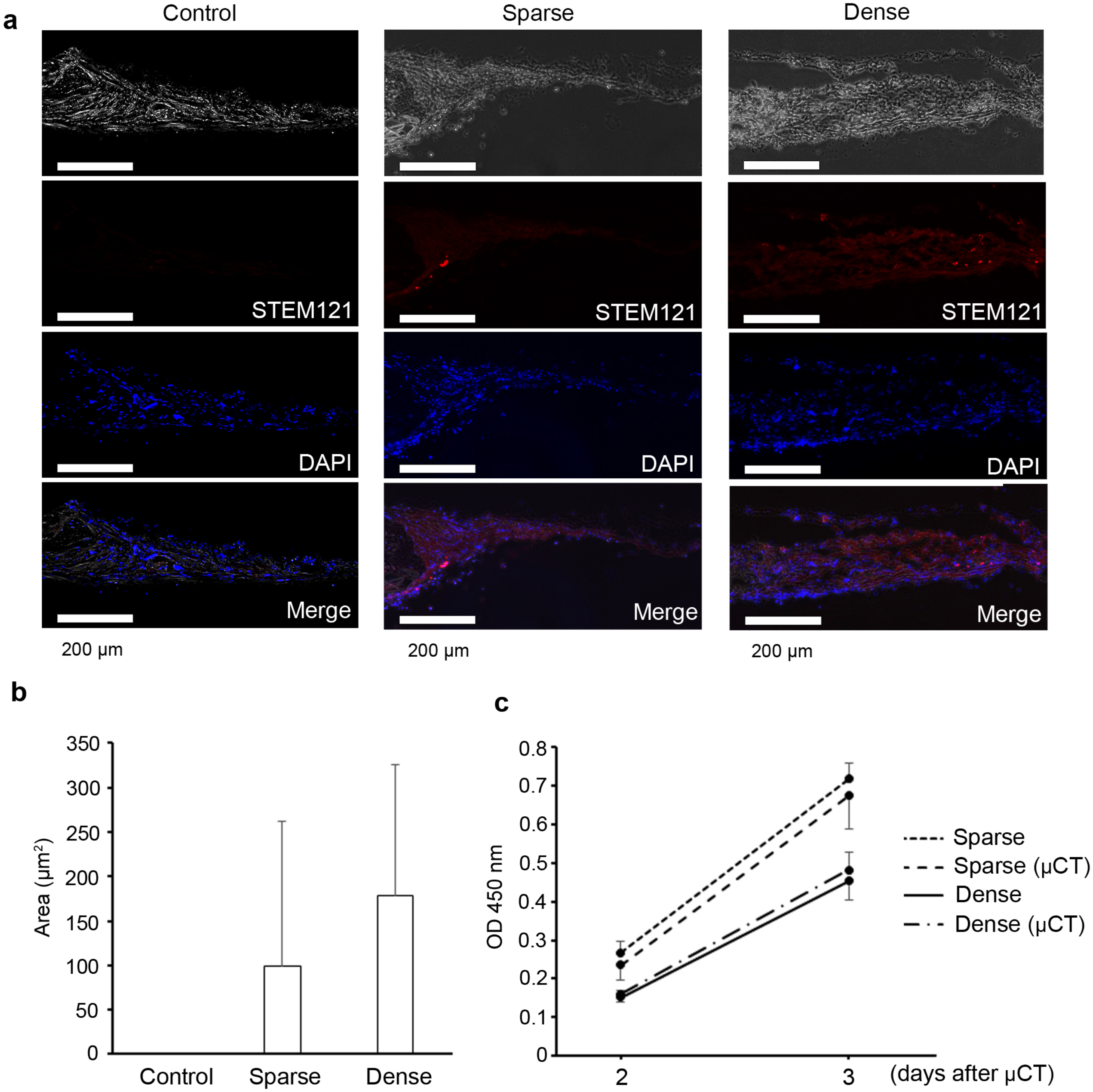

Figure S4. Stem121 immunohistochemical staining and cell proliferation assay after micro-CT scanning. (a) Representative images of Stem121⁺ cells in each group. (b) Stem121⁺ area was measured by Image J software. There was no significant difference in the Stem121⁺ area between sDPSC-transplanted and dDPSC-transplanted groups (F-test and Student's *t*-test, n = 4 images). (c) Cell proliferation assay after micro-CT scanning revealed that radiation by micro-CT did not affect the cell proliferation of DPSCs (Mann-Whitney U test, n = 7).

Fig. S5

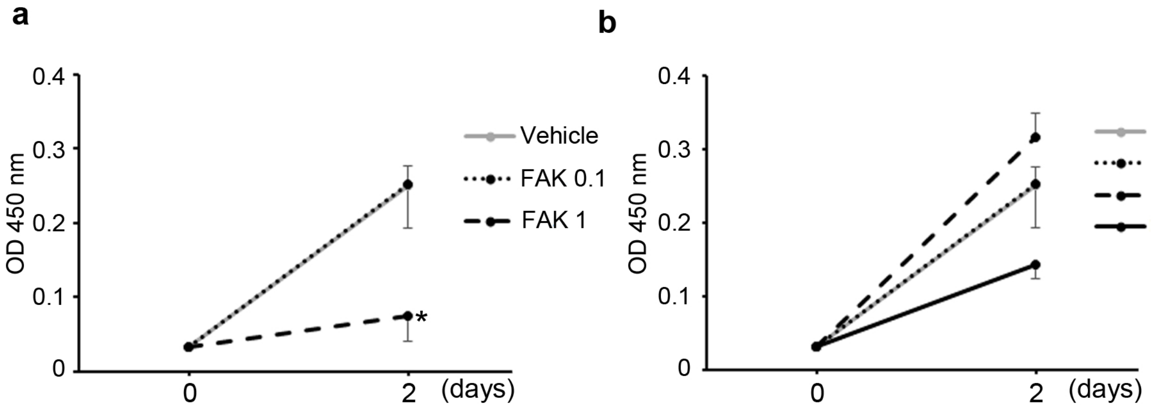

Figure S5. Proliferation assay under the presence of inhibiters. (a) FAK inhibiter 14 (Vehicle; DMSO, inhibiter; 0.1 and 1 μM) and (b) LY294002 (Vehicle; DMSO, inhibiter; 0.1, 1 and 10 μM) induced no harmful effect on cell proliferation except high concentration (1 μM) of FAK inhibiter 14 *: $p = 0.0164$ (Kruskal–Wallis test with post-hoc Dunnett's multiple comparison test). The error bar is SD ($n = 7$).
